# Financial and Treatment Compliance Challenges Among Diabetic Patients: A Cross-Sectional Study From Pakistan

**DOI:** 10.7759/cureus.78856

**Published:** 2025-02-11

**Authors:** Usman Zafar, Muhammad Suleman, Moiz N Butt, Sundas N Butt, Talha Akhtar Barra, Ahmed Faraz, Muhammad Zahid Siddique, Muhammad Aleem, Ashir Iqbal, Farrukh Ansar

**Affiliations:** 1 Department of Medicine, Alkhidmat Raazi Hospital, Rawalpindi, PAK; 2 Department of Medicine, Dr. Akbar Niazi Teaching Hospital, Islamabad, PAK; 3 Department of Emergency Medicine, Cambridge University Hospitals, Cambridge, GBR; 4 Department of Social Sciences and Humanities, National University of Sciences and Technology (NUST) School of Social Sciences and Humanities, Islamabad, PAK

**Keywords:** diabetes mellitus, economic burden of diabetes, healthcare costs in low-resource settings, socio-economic impact of diabetes, treatment compliance in diabetes

## Abstract

Introduction

Diabetes mellitus is a rapidly growing global health challenge, with significant health, economic, and social implications. This chronic condition imposes substantial financial burdens on individuals and healthcare systems, particularly in low-resource settings. This study aimed to assess the financial burden and treatment compliance among adult diabetic patients attending a tertiary care hospital in Rawalpindi, Pakistan.

Methods

A cross-sectional survey was conducted at the Diabetes Clinic of Alkhidmat Raazi Hospital, Rawalpindi. Data were collected using a structured questionnaire from 307 diabetic patients aged ≥20 years. The questionnaire captured demographics, socio-economic factors, direct medical costs, and treatment compliance. Data were analyzed using IBM SPSS Statistics for Windows, Version 23 (Released 2015; IBM Corp., Armonk, New York, United States).

Results

The mean annual direct medical cost was 55,113 PKR (USD 270), with medication expenses contributing significantly. Non-adherence to treatment was prevalent, with 129 (42%) patients citing financial constraints as the primary barrier. Productivity losses were reported by 234 (76%) participants, with an estimated 25% reduction in work output among affected individuals. Despite these challenges, 258 (84%) of participants reported moderate satisfaction with their health.

Conclusion

Diabetes imposes a substantial economic burden on patients in Pakistan, exacerbated by limited healthcare support and financial constraints. Addressing these challenges through policy reforms and targeted interventions is imperative to improve patient outcomes and alleviate the socio-economic impact of the disease.

## Introduction

Diabetes mellitus, a chronic metabolic disorder, remains one of the fastest-growing global health emergencies of the 21st century, posing significant health, social, and economic challenges worldwide [1]. Diabetes continues to impose a growing global burden, affecting individuals, families, and nations at an alarming rate. According to the IDF Diabetes Atlas (2021), 10.5% of the global adult population (20-79 years) currently lives with diabetes, with nearly half of these individuals unaware of their condition. This hidden epidemic underscores the urgent need for widespread awareness and early detection. Projections for 2045 indicate that diabetes prevalence will rise dramatically, affecting one in eight adults (approximately 783 million), marking a staggering 46% increase from current levels [2]. Tragically, diabetes-related causes account for over one million deaths annually, underscoring the critical need for urgent public health interventions [3].

The Middle East and North Africa (MENA) region bore the highest prevalence of diabetes globally in 2019, with 12.2% of the adult population affected [4]. Alarmingly, the region is projected to experience a 96% increase in diabetes prevalence by 2045, making it one of the most significantly impacted areas worldwide, second only to the African region, which faces a projected rise of 143% [4]. In contrast, the increase in diabetes prevalence over the same period is expected to be comparatively modest in Europe (15%) and North America/Caribbean regions (33%) [5]. These disparities highlight the urgent need for targeted interventions in high-burden regions like MENA to mitigate the growing diabetes epidemic [5]. In Pakistan, the prevalence of diabetes has reached epidemic proportions [6]. According to the IDF, in 2022, 26.7% of adults in Pakistan were living with diabetes, translating to an estimated 33 million cases [2].

The economic burden of diabetes is significant, with an estimated cost of 827 billion USD per annum globally [7]. Literature shows that in low-income countries, the average cost is USD 242 per individual, while in high-income countries, it soars to USD 10,801. Direct medical expenses constitute the largest share of these costs, comprising 56% in low-income nations and 74% in high-income countries [8]. The total annual (direct medical and non-medical and indirect) costs for diabetes care in South Asia range from $483 to $2637 per patient [9]. Non-medical costs are driven by factors such as travel expenses, accommodation, lost productivity, caregiver support, medical supplies, home modifications, and psychosocial or legal expenses [9]. The financial toll of diabetes extends beyond direct medical costs, affecting individuals, families, and entire healthcare systems. It has been estimated that the global direct health expenditure on diabetes will reach 1.03 trillion USD by 2030 and 1.05 trillion USD by 2045 [10].

Pakistan, classified as a lower-middle-income country, faces significant challenges in its healthcare sector. Despite efforts to improve access to healthcare, a large portion of the population still struggles with limited access to essential medical services. The healthcare system is often underfunded, with inadequate infrastructure, a shortage of medical professionals, and poor quality of care in rural and underserved areas. The country’s per capita healthcare expenditure remains low, contributing to disparities in health outcomes. Many Pakistanis rely on out-of-pocket spending for medical treatment, which further burdens the economically disadvantaged population [11]. In 2020, Pakistan's population reached 220.9 million, and the direct expenses associated with diabetes were projected at 495.0 billion PKR (1.776 billion USD) [8]. In 2007, the estimated annual direct cost of managing diabetes was approximately USD 197 per individual further illustrating the financial strain placed on affected individuals in a context where government support or health insurance is minimal or absent [12].

Given the alarming prevalence of diabetes in Pakistan and the significant economic burden it imposes, understanding and quantifying the direct medical costs per individual is crucial for effective healthcare planning and policy development. Accurate cost estimation can guide resource allocation, identify gaps in healthcare delivery, and inform strategies to alleviate the financial strain on individuals and families. This study aims to calculate the direct medical cost of diabetes per person in Pakistan, shedding light on the economic challenges faced by the population and emphasizing the urgent need for sustainable, equitable healthcare solutions to address this growing public health crisis.

## Materials and methods

Study design and setting

This cross-sectional survey was conducted in the Diabetes Clinic of Alkhidmat Razi Hospital, Rawalpindi, which is a secondary care private hospital. The study period was from June 2022 to December 2022. The study aimed to evaluate the financial burden and treatment compliance among adult diabetic patients attending the clinic.

Study population and sampling

Adult diabetic patients aged 20 years or older, irrespective of gender, were invited to participate in the study. In this study, convenience sampling was employed to select participants from a pool of patients who regularly attended the hospital’s Diabetes Clinic and were previously registered within the institution. This non-probabilistic sampling method was chosen due to its practicality in accessing individuals with well-documented medical histories, thereby enhancing the reliability and consistency of the collected data. The inclusion criteria were designed to ensure the integrity of the dataset; only patients who provided informed consent were enrolled in the study. Their comprehensive medical records included detailed documentation of past prescriptions, laboratory results, and clinical assessments, facilitating robust data analysis. Conversely, patients with incomplete medical records or those unwilling to participate were systematically excluded to minimize biases and maintain data accuracy.

Data collection instrument

A pretested, structured questionnaire was used to collect comprehensive data from participants. The questionnaire was designed to capture various aspects relevant to the study. Demographic information included details such as age, gender, educational level, occupation, and socioeconomic status. Family and medical history covered the presence of diabetes within the family, duration of the disease, and any associated comorbidities. Socio-demographic factors focused on the distance to the hospital, transportation mode, and the availability of healthcare facilities. Compliance with treatment was assessed through questions about the frequency of regular health checkups, adherence to prescribed medications, and lifestyle modifications. Additionally, the questionnaire captured direct costs associated with diabetes care, including hospital or consultant charges, medication expenses, and transportation costs. It also evaluated indirect costs, such as productivity loss, absenteeism from work, and costs incurred by caregivers. This structured approach ensured a holistic understanding of the factors influencing the financial burden and treatment compliance among diabetic patients.

As part of the study design, a pilot study was conducted on 25 participants to assess the clarity, reliability, and effectiveness of the questionnaire. Based on participant feedback and expert analysis, the questionnaire was re-evaluated and refined to enhance its comprehensibility and ensure the validity of the collected data. The reliability of the questionnaire was assessed using Cronbach’s alpha analysis, with a reliability coefficient of 0.86, indicating high internal consistency. The average inter-item covariance was 2.12.

Data collection procedure and data analysis

Trained data collectors administered the questionnaire during patient visits to the Diabetes Clinic. Information was gathered through face-to-face interviews to ensure accuracy and completeness. Confidentiality was maintained throughout the study by anonymizing participant data.

The collected data were entered and analyzed using IBM SPSS Statistics for Windows, Version 23 (Released 2015; IBM Corp., Armonk, New York, United States). Descriptive statistics were employed to summarize the dataset comprehensively. Categorical variables were presented as frequencies and percentages to provide a clear understanding of distribution patterns. For continuous variables, data were summarized as mean ± standard deviation (SD) for those with a normal distribution, while median and interquartile range (IQR) were used for variables that did not follow a normal distribution. This approach ensured precise and meaningful representation of the data, facilitating accurate interpretation and analysis.

The association between socio-demographic factors and treatment compliance was assessed using chi-square tests for categorical variables and independent t-tests or Mann-Whitney U tests for continuous variables, depending on data distribution. The statistical analysis yielded a p-value of 0.5 with a 95% confidence interval (CI), indicating no statistically significant association between the examined variables.

Ethical considerations

The study was approved by the ethical review board of Al-Khidmat Raazi Hospital, Rawalpindi, Pakistan. Written informed consent was obtained from all participants, ensuring they were aware of the study’s purpose, procedures, and their rights to withdraw at any time without consequences.

## Results

A total of 307 participants were included in the study, of whom 106 (34.5%) were male and 201 (65.5%) were female. The mean age of the participants was 52.3 ± 10.6 years. The median duration of diabetes among the participants was seven years, with the mean age at diagnosis reported as 45 ± 10.96 years. On average, patients visited their doctor twice annually, with 75 (24.4%) reporting no doctor visits in the past year, 40 (13%) making one annual visit, 77 (25%) visiting twice per year, and 61 (20%) consulting their doctor once every three months.

Socio-demographic data

Table 1 outlines the socio-demographic characteristics of the participants. Most were aged between 53 and 62 years (33.2%), with 259 (84.36%) being married. Educational status varied, with 75 (24.59%) being uneducated, 50 (16.28%) completing undergraduate education, and 189 (59.28%) holding graduate or higher degrees. Regarding income, 160 (55.9%) participants had a monthly income between 30,000 and 60,000 PKR, while 94 (32.8%) earned less than 30,000 PKR.

**Table 1:** Socio-demographic data of study participants

Description		Frequency (Percentages)
Age category (years)	22-32	6 (1.9%)
	33-42	49 (15.9%)
	43-52	89 (28.9%)
	53-62	102 (33.2%)
	63-72	50 (16.2%)
	73-82	11 (3.5%)
Marital status	Married	259 (84.36%)
	Unmarried	1 (0.33%)
	Widow	47 (15.31%)
Educational status	Not educated	75 (24.59%)
	Undergraduate	50 (16.28%)
	Graduate and above	182 (59.28%)
Income groups (monthly income in PKR)	0-30,000	94 (32.8%)
	30,000-60,000	160 (55.9%)
	61,000-100,000	24 (8.3%)
	100,000-200,000	8 (2.8%)

Ninety percent of the participants exclusively consulted private doctors for diabetes management. The mean doctor consultation fee was 1350 PKR (USD 6.6) ± 975, with a median of 1000 PKR (USD 4.9) and a range of 450 to 5000 PKR (USD 2.20-24.5). The average clinic waiting time was 2.4 hours. Monthly medication expenses averaged 2521 PKR ± 1917 (USD 12.3 ± 9.3). Among the 108 participants using insulin, the mean monthly cost of insulin was 2510 PKR ± 1520 (USD 12.3 ± 7.4).

Patient characteristics and social factors

The age at onset of diabetes is presented in Table 2. The most common age group for disease onset was 31-50 years (64.5%). A delay in initiating regular treatment after diagnosis was reported by 51 (25.4%) of participants, with 25 (12.4%) delaying treatment for more than one year. Regular doctor visits, when recommended, were not adhered to by 170 (55.3%) participants, with the primary reasons being financial constraints N = 85 (42.2%) and carelessness N = (12.9%). A significant portion of participants (56.6% male and 39.8% female) adhered to regular visits. Participants faced minimal difficulties in performing routine tasks, with only eight (3.9%) reporting challenges. The analysis of perceptions about satisfaction with personal health revealed that the majority of respondents, 258 (84.04%) individuals, reported being moderately satisfied (scores 4-7). A smaller proportion, 45 (14.6%) individuals, indicated high satisfaction with their personal health (scores 8-10). Meanwhile, only five (1.6%) individuals expressed dissatisfaction (scores 1-3).

**Table 2:** Patient characteristics and associated social factors

Description		Female (N = 201)	Male (N= 106)	Total
Starting age of disease (years)	0-10	0	1	1
	11-20	1	1	4
	21-30	14	6	23
	31-40	59	26	88
	41-50	77	36	110
	51-60	38	22	57
	61-70	11	10	19
	71-80	1	4	5
Delay in starting the regular treatment after diagnosis (years)	0	150 (74.6%)	80 (75.4%)	230
	1	26 (12.9)	14 (13.2)	40
	>1	25 (12.4)	13 (12.26)	38
No regular visit to the doctor when recommended		124	46	170
Reasons for not visiting clinic/hospital despite feeling the need	Carelessness	26 (12.9)	10 (9.43)	36
	Financial constraints	85 (42.2)	39 (36.79)	124
	Other	3 (1.49)	1 (0.94)	3
	Unavailability of an attendant	8 (3.9)	1 (0.94)	10

Financial impact

The financial burden of diabetes is summarized in Table 3. The mean direct annual cost per participant was 55,113 PKR ± 48,427 (USD 270 ± 237), while indirect costs added further strain. The total annual cost was significant, reflecting the substantial economic impact of the disease. Loss of productivity was reported by 234 (76%) of participants, with 124 (40%) estimating a 25% reduction in productivity. Annual monetary losses from productivity varied, with 96 (33.5%) reporting losses between 5001 and 10,000 PKR (USD 24.5 to 49).

**Table 3:** Financial impact of type 2 diabetes mellitus on patients

Variable	Direct Cost/ Annum	N (%)
Loss of productivity (patient perception) N = 307	0%	73 (23.7%)
	10%	98 (31.9%)
	25%	124 (40.3%)
	50% or above	12 (3.9%)
Annual monetary loss of productivity (N = 286)	0	73 (25.5%)
	less than 5000	80 (27.9%)
	5001-10000	96 (33.5%)
	10001-37500	37 (12.9%)
Financial cost Mean ± SD	55113 ± 48427 (USD 270 ± 237)	

## Discussion

This study provides critical insights into the socio-demographic characteristics, treatment adherence, and financial burdens experienced by patients with diabetes, with implications for public health and clinical management.

The sample predominantly consisted of married individuals (N= 259, 84.36%), with the majority aged between 43 and 62 years (N = 191, 78.3 %) and achieving a graduate or higher education level (N = 182, 59.28%). These demographic features align with previous studies emphasizing the rising prevalence of diabetes among middle-aged and older adults [13]. A notable finding is that 51 (25.4%) of participants delayed initiating regular treatment, with financial constraints being the leading cause (42.2%). This finding corroborates earlier research linking delayed diabetes management to financial barriers in low-to-middle-income countries (LMICs) [14].

Furthermore, adherence to regular doctor visits varied significantly, with men (56.6%) more compliant than women (39.8%). Cultural norms and gender roles may partially explain this discrepancy, as women in LMICs often prioritize household expenses over healthcare [15]. This aligns with findings that gender disparities in healthcare access and treatment adherence persist due to structural and social determinants [16]. Implementing targeted interventions to address these disparities could enhance treatment compliance and outcomes.

Globally, the economic impact of diabetes has been extensively documented, with LMICs enduring most of the financial strain. The current study highlights the profound financial burden associated with diabetes, with an average annual direct cost of 55,113 PKR ± 48,427 (USD 270 ± 237), and substantial indirect costs due to productivity loss. Approximately 40.3% of participants reported a 25% reduction in productivity, reflecting the dual economic and physical toll of diabetes. Global research indicates a stark disparity in healthcare costs between income groups, with the average cost per individual being approximately USD 242 in low-income countries, compared to a significantly higher USD 10,801 in high-income nations [8]. A study from Karachi, Pakistan in 2007 estimated annual direct cost per individual with diabetes was USD 197 [12]. A multi-center study conducted in Pakistan and published in 2022 reported the direct cost of diabetes to be USD 646 [8]. The comparatively lower costs reported in our study can be attributed to the unique setting of this research, conducted within a trust that prioritizes providing patient services at minimal expenses. This highlights the critical role of subsidized healthcare facilities in reducing the financial burden on patients and underscores the need for similar initiatives to enhance accessibility and affordability of care for those living with chronic conditions like diabetes. In Iran, the diabetes-related health expenditure per person in 2021 was estimated at approximately USD 1,354 [17]. Comparatively, in Bangladesh, the average annual cost per patient in 2019 was reported to be USD 865 [18]. A study conducted in India reported that the annual mean cost per patient ranged between 6,000 and 10,000 Indian Rupees (approximately USD 72 to 120) [19]. India's lower cost of diabetes management compared to Pakistan and Bangladesh may be attributed to its robust domestic pharmaceutical industry, which produces affordable generic medications and insulin, significantly reducing treatment expenses [20]. Government initiatives like the National Programme for Prevention and Control of Non-Communicable Diseases and Jan Aushadhi provide subsidized care, while economies of scale due to India's large diabetic population further drive down costs [21]. In contrast, Pakistan and Bangladesh rely heavily on imported drugs and lack a well-developed pharmaceutical sector, leading to higher treatment costs [22]. Additionally, Pakistan faces limited public healthcare infrastructure and relies heavily on private healthcare, which increases out-of-pocket expenses for patients [23]. The dominance of private healthcare exacerbates the financial burden, making diabetes management significantly more expensive compared to India.

The reported monthly medication costs (2521 PKR ± 1917) and insulin expenses (2510 PKR ± 1520) reveal an inequitable access pattern, where 90% of participants exclusively consult private doctors. This over-reliance on private healthcare, often associated with higher out-of-pocket expenses, exacerbates financial hardships. An analysis of total out-of-pocket expenditure reveals that 81% was allocated to private-sector healthcare services, while only 19% was incurred by users of public health facilities [23]. This reflects Pakistan's longstanding pattern of inadequate investment in healthcare, with public health expenditure accounting for less than 1% of GDP and under 5% of total government spending [23]. There is a need for subsidized medications and publicly funded health systems to alleviate economic strain.

Despite financial and health challenges, 84.04% of respondents reported moderate satisfaction with personal health. This could reflect cultural attitudes toward health or resilience in coping mechanisms. However, this satisfaction contrasts with the substantial productivity loss and non-compliance rates. Future qualitative studies could explore the psychological factors influencing this paradox, aligning with earlier work by Chen et al. [24] which identified resilience as a mediator in chronic disease management.

Notable gender differences were observed in treatment adherence and productivity loss, with men demonstrating higher adherence but experiencing greater financial impacts. These findings are consistent with prior research showing that men often receive preferential healthcare resources in patriarchal societies [25]. Socioeconomic disparities were also evident, as 32.8% of participants belonged to the lowest income group (<30,000 PKR monthly) while 55.9% were earning between 30,000 and 60,000, significantly influencing their healthcare access and adherence. Addressing these disparities requires targeted community-based interventions and policies to ensure equitable healthcare access.

The findings underscore the urgent need for health policy reforms in Pakistan. Subsidized medications, enhanced public healthcare infrastructure, and community health education can mitigate the barriers identified in this study. Additionally, integrating diabetes care into primary healthcare settings could improve accessibility and affordability. Future research should focus on longitudinal analyses to examine the long-term effects of financial constraints and treatment adherence. Qualitative studies could also provide deeper insights into patient perceptions and decision-making processes, complementing quantitative findings.

This study has several limitations that should be acknowledged. Firstly, its cross-sectional design restricts the ability to establish causal relationships between variables, as it captures data at a single point in time. Secondly, the use of convenience sampling may introduce sampling bias, potentially limiting the generalizability of the findings beyond the study population. Additionally, the reliance on self-reported data raises the possibility of recall bias, particularly in estimating productivity loss, healthcare expenses, and treatment adherence, as participants may not accurately remember past healthcare utilization or financial expenditures. Furthermore, while efforts were made to enhance data accuracy by cross-referencing medical records for laboratory results and medication history, observer bias may still be present in subjective assessments. Despite these limitations, this study provides valuable insights into the healthcare and economic burden of diabetes, contributing to the development of targeted interventions aimed at improving diabetes management and patient outcomes. Future studies employing longitudinal designs, objective productivity loss measures, and randomized sampling are recommended to enhance the robustness of findings.

## Conclusions

This study highlights the significant challenges faced by individuals with diabetes, including financial burdens, treatment non-adherence, and socio-demographic disparities, which collectively impact their quality of life and productivity. The findings emphasize the urgent need for accessible and affordable healthcare services, particularly for low-income groups, and targeted interventions to address delays in treatment initiation and regular follow-ups. Addressing these barriers through public health reforms, subsidized care, and community education can significantly improve treatment outcomes and reduce the economic and health burden of diabetes, fostering a more equitable healthcare system.

## Appendices

Questionnaire: valuing the economic impact of diabetes

Demographics

1. Respondent's Age: __________________________ years

2. Respondent's Gender: __________________________ (Male/Female/Transgender)

3. Respondent's Education Level: __________________________

4. Respondent's Native Language: __________________________

5. Respondent's Marital Status:

   - Married

   - Unmarried

   - Other: __________________________

Household details (including parents/spouse/children/siblings)

No

Relation

Age

Education (in years)

Economic status

Diabetic?

Duration of diabetes

Cause of Death (if deceased)

Diabetes-Related Information

6. How long have you been diagnosed with diabetes? __________________________ years

7. In your opinion, how severe is diabetes?

   - Mild

   - Moderate

   - Severe

8. How long have you been receiving regular treatment for diabetes (e.g., using medication)? __________________________ years

9. How often do you visit your healthcare provider for diabetes management? __________________________ times (Monthly/Annually)

10. Type of healthcare provider you consult: __________________________ (Private/Public)

11. Average consultation fee per visit: __________________________ currency

12. Average time spent per visit (including travel and waiting): __________________________ hours

13. Transportation cost per visit (fare or fuel): __________________________ currency

14. Food expenses during the visit: __________________________ currency

15. Monthly cost of diabetes medication: __________________________ currency

16. Do you take medication consistently?

    - Yes

    - No

    If No, specify the reason:

       - Negligence

       - Cost of medication

       - Lack of accessibility to medication

       - Other: __________________________

17. Monthly cost of insulin (if applicable): __________________________ currency

18. Frequency of insulin device replacement: __________________________ (Months/Years)

19. Cost of insulin device: __________________________ currency
